# Automatic Modal Parameter Identification for Offshore Wind Turbines Using Modified Clustering-Based Methodology

**DOI:** 10.3390/s26082536

**Published:** 2026-04-20

**Authors:** Yang Yang, Fayun Liang, Qingxin Zhu, Hao Zhang

**Affiliations:** 1 Engineering Research Center of Offshore Wind Technology Ministry of Education, Shanghai University of Electric Power, Shanghai 200090, China; yangyang@shiep.edu.cn; 2 Department of Geotechnical Engineering, Tongji University, Shanghai 200092, China; 3 School of Environment and Architecture, University of Shanghai for Science and Technology, Shanghai 200093, China; zhuqingxin@usst.edu.cn; 4 College of Environmental Science and Engineering, Donghua University, Shanghai 201620, China; hzhang@dhu.edu.cn

**Keywords:** offshore wind turbines, dynamic response, automatic modal parameter identification, stochastic subspace identification, clustering algorithm, system identification, control

## Abstract

**Highlights:**

**What are the main findings?**

- A novel modal parameter identification method with modified clustering analysis was proposed.
- The proposed method possesses the accuracy required for automated modal parameter identification.

**What are the implications of the main findings?**

- The method is applicable to the modal characterization of offshore wind turbines.
- The study provides technical support for the diagnosis of abnormal states of offshore wind turbines.

**Abstract:**

Offshore wind power stands as a clean and low-carbon energy option that is booming as part of the efforts to achieve the goal of carbon neutrality. Effectively monitoring the dynamic response of wind turbines is a necessity to analyze the modal parameters, which are key parameters to assess whether the wind turbines are operating safely. Modal parameter identification for offshore wind turbines (OWTs) becomes essential through analyzing the dynamic response, given the limited acceptable range of natural frequencies under dynamic loads. This paper introduces a novel machine learning-based method that combines the SSI-data (data-driven stochastic subspace identification) modal parameter identification method with clustering analysis, employing DBSCAN (Density-Based Spatial Clustering of Applications with Noise) and the K-means cluster algorithm. The proposed method can automatically define the number of K-means clusters. The validation was carried out through a theoretical analysis using a four-degree-of-freedom model and Opensees numerical simulation model of an OWT. The verification and case study outcomes demonstrate that the proposed method possesses the accuracy required for automated modal parameter identification. Compared with the benchmark case results, the differences between the frequencies identified by the proposed method and the reference values are 0.0%, 0.30%, and 0.18% for the first three orders, respectively. This research not only provides valuable insights for professionals in related dynamic monitoring fields but also offers technical support for diagnosing abnormal states of OWTs utilizing dynamic response data.

## 1. Introduction

The offshore wind power industry is rapidly rising, and the global installed capacity of OWTs reached 83.91 GW by the end of 2024, as wind energy resources are abundant, clean, and low-carbon [1]. OWTs operate in harsh environments, enduring dynamic loads caused by wind, waves, and currents, as well as operational excitation and potential seismic loads [2,3]. The degradation of foundation performance and weakening of soil strength [4,5,6] on the pile side may lead to changes in the dynamic performance of wind turbine structures, posing a threat to their operational safety [7]. The dynamic performance of wind turbine structures can be characterized by modal parameters [8], making them significant for diagnosing the condition of wind turbine structures [9,10].

Currently, the identification of modal parameters in operational wind turbines heavily relies on the collected dynamic monitoring data from sensors installed on the wind turbine towers [11]. The commonly used stochastic subspace identification (SSI) algorithm is employed for modal parameter identification [12,13]. However, due to the impact of environmental noise such as dynamic loads induced by wind, waves, and currents, as well as operational excitations of wind turbines, the presence of false modes is inevitable. Additionally, different modes may have very close frequencies, making it challenging to distinguish them in measurement data, leading to the emergence of false modes [14]. The existence of these false modes results in identification errors in the process of modal parameter identification. To address this issue, more intelligent and efficient methods are needed [15].

Modal parameter identification research has benefited from the successful application of machine learning techniques [16]. For example, Devriendt et al. applied an automatic monitoring approach to identify and track the resonant frequencies and damping values of an OWT, but the proposed method is suitable for wind turbines under parked operating conditions [17]. Li et al. proposed a modal parameter identification procedure for high arch dams utilizing clustering algorithms [18]. The method cannot automatically identify the cluster centers to extract modal parameter information. Yang et al. used unsupervised learning using K-means clustering to identify different-order modal parameters from stabilization diagrams [19]. Devriendt et al. adopted fuzzy clustering algorithms to automate the modal parameter identification which were efficient in the identification and tracking of wind turbine models [20]. However, K-means and fuzzy clustering algorithms require setting the number of clusters in advance, which may limit their automatic modal parameter identification capabilities. While some researchers [18,21] have applied the DBSCAN clustering method for modal parameter identification in structures such as bridges [22,23] and helicopter blade modal analysis [24], DBSCAN does not require defining the number of clusters and is a density-based clustering algorithm. Nevertheless, this method involves setting different distance and neighborhood sample number thresholds, which significantly affect the final clustering results and may lead to an unreasonable number of clusters. Points from the stabilization diagram of the same mode can be divided into different clusters if they appear discontinuously. The identification and data analysis capabilities for modal parameters of OWTs during lifetime lag behind the data acquisition capabilities [25]. Effectively managing the vast amount of data obtained from offshore wind farms and achieving automatic and efficient identification of modal parameters to enable faster and more accurate diagnosis of wind turbine operational states are the main challenges faced in this field [26]. Automatic identification is a key issue in this context. However, the current methods for modal parameter identification in wind turbines need improvement and enhancement. Based on the extended analysis and discussion, to gain a better understanding of the current state of research on the combination of clustering methods and SSI, the existing key achievements and critical limitations are systematically summarized in Table 1.

This paper focuses on the research question of automatic and accurate modal parameter identification for wind turbine support structures. Aiming to improve the automation of modal identification, the main objective is to propose a novel machine learning-based method by improving the existing approach [31], while several significant modifications have been implemented to enhance its automatability. It primarily employs the SSI-data modal parameter identification method to obtain stabilization diagram, and then carry out false mode removal, initial clustering using DBSCAN, noise reduction, and improved K-means clustering to automatically identify the modal parameters of wind turbines. The main contributions of this study can be summarized as follows: (1) The proposed method combines the advantages of DBSCAN and K-means clustering algorithms, and aims to solve the automatic identification problem of modal parameters for OWTs. (2) This machine learning-based approach optimizes the traditional clustering algorithm by eliminating the need to specify the number of clusters and efficiently achieves automatic modal parameter identification for OWTs. (3) The effectiveness and accuracy of the method are verified through both theoretical validation and application to a real offshore wind farm project for the automatic identification of modal parameters by comparing the results with FDD (Frequency Domain Decomposition) methods [32]. This study provides technical support for the diagnosis of abnormal states of OWTs and serves as a reference for relevant regulations and standards for the assessment of OWT service states.

To clearly present the research framework of this paper, the remainder of the manuscript is organized as follows. Section 2 elaborates on the methodology, including an introduction to the modified clustering-based approach (Section 2.1) and a sequential explanation of the improved methodology (Section 2.2). Section 3 presents the research results, which are divided into three parts: theoretical condition analysis (Section 3.1), automatic modal parameter identification of the simplified structure (Section 3.2), and modal identification based on numerical modeling results (Section 3.3). Section 4 conducts in-depth discussion, involving benchmark dataset-driven comparative analysis (Section 4.1) and real-world OWT implementation with FDD method comparison (Section 4.2). Finally, Section 5 summarizes the entire study and puts forward relevant conclusions.

## 2. Methods

The modal parameter identification methodology for offshore wind turbines will be introduced in this section, intended to provide a comprehensive and accessible framework. The section begins with an introduction to the improved method founded on modified clustering, followed by a step-by-step elaboration of the revised procedure.

### 2.1. Data-Driven Stochastic Subspace Identification (SSI-Data)

The input–output relationship of a linear time-invariant system is described using the state-space model:

(1) $$X_{k+1}=Ax_{k}+Bu_{k}\;y_{k}=Cx_{k}+Du_{k}+e_{k}^{m}$$

where $x_{k}$ is the state vector of the system, $y_{k}$ is the output vector, $u_{k}$ is the input vector, $e_{k}^{m}$ is the measurement error, and A, B, C, and D are the system matrices. Herein, only the outputs $y_{k}$ are observed. $u_{k}$ and $e_{k}^{m}$ are assumed to be discrete white noise random processes; thus, the model can be rewritten as

(2) $$\begin{array}{l} X_{k+1}=Ax_{k}+w_{k}\\ y_{k}=Cx_{k}+v_{k} \end{array}$$

where $w_{k}$ and $v_{k}$ are the process noise and output noise, respectively.

The SSI-data algorithm aims at identifying the system matrices A and C. This method starts by collecting the outputs into a Hankel matrix [33,34], which is defined as

(3) $$Y_{O/2i-1}=\frac{Y_{p}}{Y_{f}}=\left(\frac{Y_{O/i-1}}{Y_{i/2i-1}}\right)$$

Herein, the row space of the future outputs ($Y_{f}$) is projected into the row space of the past outputs ($Y_{p}$). Then, the QR factorization is implemented to the data Hankel matrices.

(4) $$Y_{O/2i-1}=RQ^{T}=\left(\begin{array}{cc} R_{11} & 0\\ R_{21} & R_{22} \end{array}\right)\left(\begin{array}{c} Q_{1}^{T}\\ Q_{2}^{T} \end{array}\right)$$

The projections of the future row spaces to the past row spaces can be obtained:

(5) $$O_{i}=Y_{f}/Y_{p}=Y_{f}Y_{p}^{T}{\left(Y_{p}Y_{p}^{T}\right)}^{+}Y_{p}=R_{21}Q_{1}^{T}$$

The main theorem of the stochastic subspace identification states that the projection $O_{i}$ can be factorized as the observability matrix Ti and the Kalman filter state sequence $\overset{\wedge }{X}$.

(6) $$O_{i}=T_{i}\overset{\wedge }{X}$$

The output matrix C equals the first l rows of the observability matrix Ti:

(7) $$C=T_{i}\left(1:l,:\right)$$

Let matrix $\overset{—}{T}$ equal the last l rows of Ti and $\underset{—}{T}$ equal the first l rows of Ti. Accordingly, the system matrix A can be obtained as

(8) $$A={\underset{—}{T}}^{+}\overset{—}{T}$$

The modal parameters (eigenfrequencies, damping ratios and mode shapes) can be calculated from the system matrices A and C.

### 2.2. Introduction to the Methodology Based on Modified Clustering

**Figure 1:**
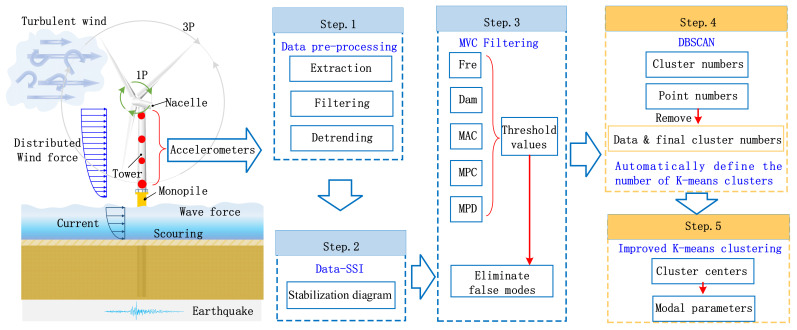
Automated modal parameter identification method based on modified clustering.

Modal parameters can reflect the dynamic characteristics of OWTs. Given the narrow range of inherent frequencies in wind turbine support structures, it is of vital significance to identify modal parameters to ensure the safe operation of OWTs. This paper presents an automated modal parameter identification method based on modified clustering suitable for OWTs structures, as shown in Figure 1. The automatic modal parameters identification method begins with data pre-processing. Next, it utilizes SSI-data to obtain a stabilization diagram. It employs an initial filtering process based on modal validation criteria (MVC) [35]. The above steps are similar to the previous study conducted by Mao et al. [31], while several significant modifications have been implemented in the following steps. Subsequently, the DBSCAN algorithm is applied for clustering, with the removal of noisy data, thereby determining the number of clusters for the subsequent K-means clustering. Lastly, an improved K-means clustering technique is utilized to group data points from the same cluster, obtaining cluster centers and thereby acquiring frequency information. The novelty of the modified approach is that it eliminates the need to specify the number of clusters and automatically achieves modal parameter identification for OWTs. The validation of this method will be discussed in Section 3, and the results demonstrate that this approach exhibits excellent robustness and ease of operation.

### 2.3. Sequential Explanation of the Modified Methodology

To offer a more comprehensive introduction to the modified methodology, this section elaborates on each step of the methodology. It encompasses both the previously presented method [31] and the modification made in this study. Each procedure is carefully presented to ensure a clear and coherent understanding, enabling readers to follow the logical sequence of the steps without confusion. This detailed and sequential introduction aims to offer a comprehensive and easily accessible guide based on the visual representation in Figure 1. The method consists of the following steps:

(1) Data pre-processing

The monitoring data is first processed, including data extraction, filtering and data detrending. Since the data is continuous, a specific time segment is selected for data extraction. If the sampling frequency is excessively high, it can be appropriately reduced. Butterworth filtering is applied to remove noise and extract the desired frequency range. If any baseline drift exists in the data due to equipment reasons, detrending is performed.

(2) SSI-data for obtaining stabilization diagram

**Figure 2:**
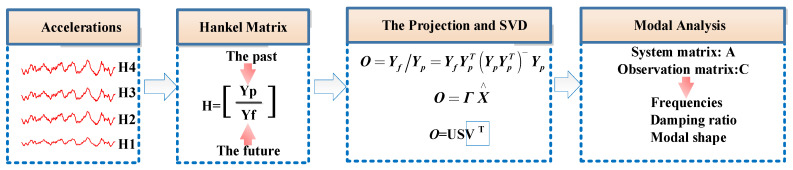
SSI-data modal parameter identification process.

The data processed as described above is subjected to SSI-data modal parameter identification. This analysis aims to determine wind turbine modal parameters, including frequency, damping, and mode shapes, as shown in Figure 2.

(3) Initial MVC filtering

In the modal parameter analysis process, due to modal overlap, numerical errors, measurement noise, and other factors, false modes are present in the stabilization diagram obtained by the aforementioned SSI-data. It is necessary to eliminate these false modes. For each computed vibration mode vector, frequency difference (Fre), damping ratio difference (Dam), mode assurance criterion (MAC), modal phase coherence (MPC), and mean phase difference (MPD) values are calculated. Threshold values for each parameter are determined, and modes meeting the specified conditions are retained, while modes failing to meet the threshold selection criteria are discarded.

(4) DBSCAN to obtain clustering numbers

In this step, the order and mode data of the data that has undergone the initial MVC filtering are first normalized, while frequency is not normalized, to facilitate the rapid extraction of frequency information for different modes in subsequent steps. The numerical gap between different orders would artificially inflate the distance weight of modal order in DBSCAN calculations. For this reason, the modal order data were normalized. In contrast, frequencies corresponding to modal order possess clear physical significance and do not require normalization, because this would distort the natural physical scaling relationships between different modes. Clustering is performed based on DBSCAN, which enables the automatic acquisition of the number of clusters and the number of data points in each cluster, and is a major innovation point of this paper. First, isolated noise points are removed based on DBSCAN clustering, with neighborhood radius as 0.0805 and minimum number of points required to form a core point as 4. Isolated points that cannot form density-connected clusters are directly identified as unreasonable noise points and eliminated. Second, small clusters are removed according to a cluster-size threshold. The number of data points in each remaining DBSCAN cluster is counted, and a cluster is explicitly defined as “unreasonable” if its size falls below a predefined minimum threshold. In this study, the threshold is set to 8, determined based on the statistical distribution of the modal identification data and the physical characteristics of structural modes. Real structural modes generally form dense, stable clusters with sufficient sample sizes, whereas unreasonable clusters are sparse and contain only a small number of points. The number of clusters and data points within the range of interest frequencies are extracted, and unreasonable data points and clusters are removed. Finally, a reasonable number of clusters is automatically output for further analysis.

(5) Improved K-means clustering

Automatically define the number of K-means clusters as the clustering quantity determined in step (4)’s final output. Perform K-means clustering to obtain the values of cluster centers, representing the frequency, damping ratio, and mode shape of each mode. Noted that the mode shape can be generated in the process of clustering and it is not included in the following context.

## 3. Results

This section is a validation of the results of the aforementioned automatic modal parameter identification algorithm. Using a four-degree-of-freedom model and a numerical simulation model, the computed modal frequencies are compared against the modal parameters calculated by employing the automatic modal parameter identification method described above, confirming the accuracy of the methodology.

### 3.1. Theory Conditions

**Figure 3:**
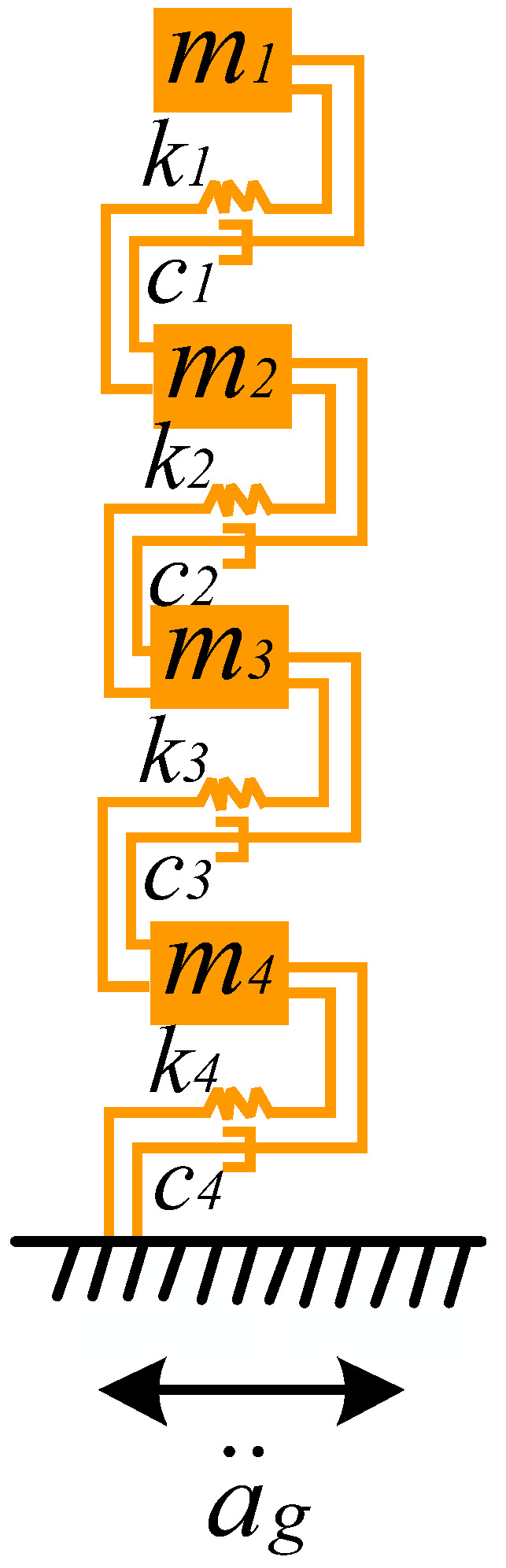
The four-degree-of-freedom model.

To verify the correctness of the automatic modal parameter identification method, a four-degree-of-freedom model was designed, as depicted in Figure 3.

The motion equations for this simplified four-degree-of-freedom structure are

(9) $$M\ddot{u}+C\dot{u}+Ku=-M\ddot{a_{g}},$$

where M is the mass matrix, $\left[\begin{array}{cccc} m_{1} & 0 & 0 & 0\\ 0 & m_{2} & 0 & 0\\ 0 & 0 & m_{3} & 0\\ 0 & 0 & 0 & m_{4} \end{array}\right]$;

C represents the damping matrix, $\left[\begin{array}{cccc} c_{1} & 0 & 0 & 0\\ 0 & c_{2} & 0 & 0\\ 0 & 0 & c_{3} & 0\\ 0 & 0 & 0 & c_{4} \end{array}\right]$;

K stands for the stiffness matrix, $\left[\begin{array}{cccc} k_{1} & -k_{1} & & \\ -k_{1} & k_{2}+k_{1} & -k_{2} & \\ & -k_{2} & k_{3}+k_{2} & -k_{3}\\ & & -k_{3} & k_{3}+k_{4} \end{array}\right]$;

$\ddot{u}$ denotes acceleration; $\dot{u}$ represents velocity; u signifies displacement; and $\ddot{a_{g}}$ is the acceleration applied at the base.

In the process of modal analysis, to obtain the eigenvalues and eigenvectors of the four-degree-of-freedom system, it is necessary to consider the system as an undamped system for free vibration. The vibration equation is given by

(10) $$M\ddot{u}+Ku=0$$

The equation for eigenvalues is

(11) $$(K-\omega^{2}M)\phi =0,$$

where ω represents the angular frequency and ϕ denotes the modal vector. From the eigenvalues and eigenvectors, the modal frequencies are directly derived as the square root of the eigenvalues, as shown by the following formula:

(12) $$\phi =\left[\begin{array}{cccc} \phi_{1} & \phi_{2} & \phi_{3} & \phi_{4} \end{array}\right],$$

With assigned masses for the four-degree-of-freedom structure as 100, 100, 100, 100 kg, and stiffness values of 800, 1600, 3200, 4800, the calculated modal frequencies using the aforementioned algorithm are: 0.277 Hz, 0.636 Hz, 1.009 Hz, 1.597 Hz.

### 3.2. Automatic Modal Parameter Identification of the Simplified Structure

**Figure 4:**
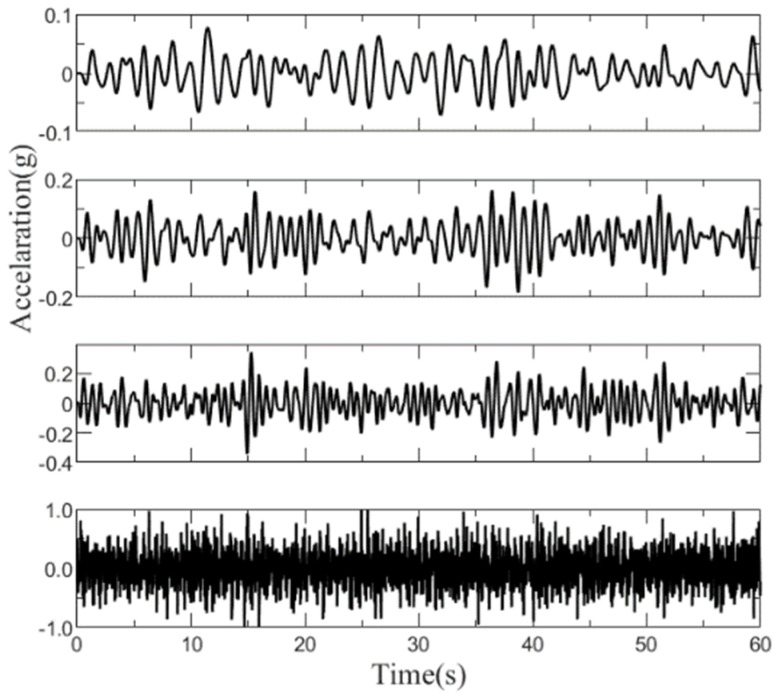
The acceleration time history results for the four masses.

White noise excitation is applied for the four-degree-of-freedom structure, and the response under white noise excitation is computed using the Newmark-β method to obtain acceleration data for four points, as illustrated in Figure 4. Utilizing this acceleration data, the structural modal frequencies are computed using the modal parameter identification algorithm.

**Figure 5:**
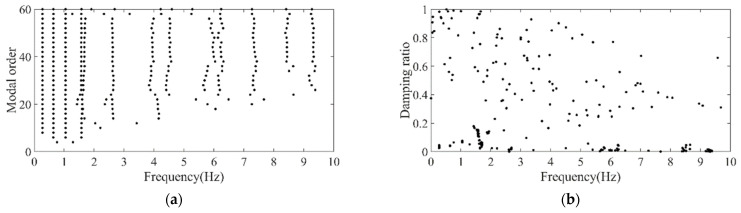
Original stabilization diagram of the simplified structure. (**a**) Model order and frequency; (**b**) damping ratio and frequency.

**Figure 6:**
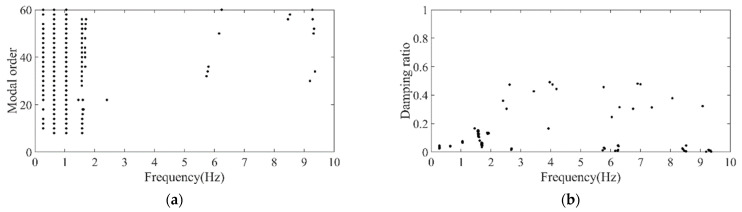
Stabilization diagram after MVC of the simplified structure. (**a**) Model order and frequency; (**b**) damping ratio and frequency.

**Figure 7:**
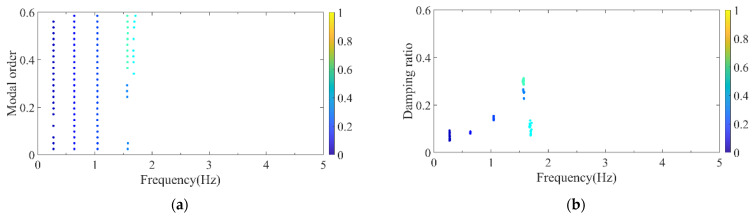
DBSCAN clustering results of the simplified structure. (**a**) Normalized model order and frequency; (**b**) normalized damping ratio and frequency.

**Figure 8:**
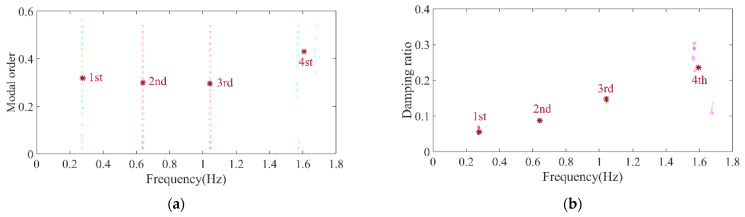
Clustering centers results of the simplified structure. (**a**) Normalized model order and frequency; (**b**) normalized damping ratio and frequency.

Firstly, the stabilization diagram was obtained using SSI-data, as shown in Figure 5. The MVC initial filtering results, as seen in Figure 6, still indicate the presence of false modes. Next, the data was normalized, and a frequency threshold of 5 Hz was set to filter out data unrelated to vibration frequencies. The 5 Hz low-pass filter threshold was selected based on the dynamic characteristics of offshore wind turbine support structures. The dominant vibration components governing the global structural response, including the first several global modal frequencies, rotor-induced 1P/3P excitation frequencies, and wave-induced vibration frequencies, are all concentrated below 2 Hz. The 5 Hz cutoff ensures the retention of structurally relevant vibration information and improves signal quality, which is consistent with common filtering practice in offshore wind structural health monitoring. DBSCAN utilized the normalized data for density-based clustering, as shown in Figure 7. It should be emphasized that the K value in K-means is not manually predefined, but automatically determined by the number of valid clusters retained after DBSCAN denoising and removal of small clusters, and this value is directly used as the cluster number for the subsequent improved K-means step. This automatic design ensures consistency between the DBSCAN pre-processing and the improved K-means clustering, and eliminates the subjectivity associated with manual selection of K. Finally, using the improved K-means clustering, the DBSCAN clustering number, both reasonable and unreasonable, was automatically identified. This number was assigned to the K-means clustering, and the data within the same cluster were further aggregated to obtain frequency information, resulting in values of 0.277 Hz, 0.639 Hz, 1.041 Hz, and 1.608 Hz, as shown in Figure 8.

**Figure 9:**
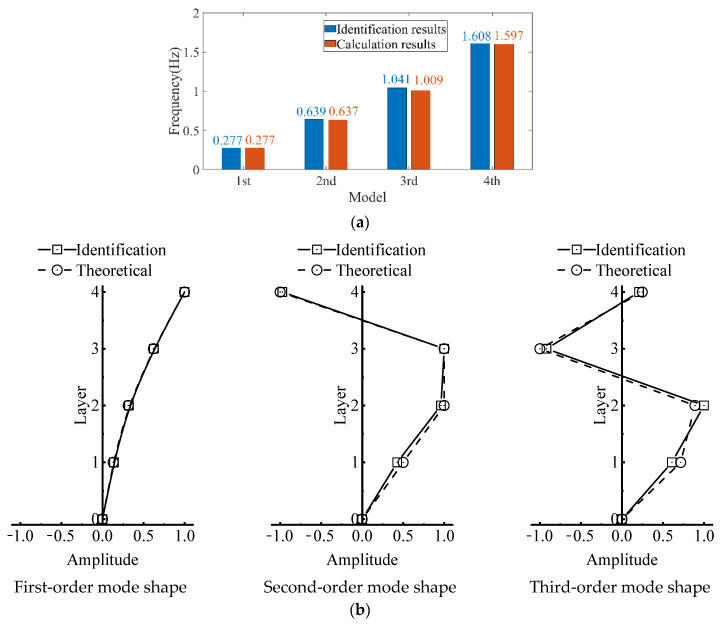
Comparation between theory and automatic modal identification results. (**a**) Frequencies. (**b**) Mode shapes.

The frequency recognition results were compared with the calculated results, as shown in Figure 9. The mean error is 0.0115 Hz, the median error is 0.007 Hz, and the maximum error is 0.032 Hz. The theoretical values and identified results of the first three mode shapes are compared in Figure 1, and the identified mode shapes agree well with the theoretical ones. In addition, Rayleigh damping is adopted in the numerical model with the formulation c = 0.05m + 0.02k. The first three natural frequencies are 0.277 Hz, 0.639 Hz and 1.009 Hz respectively. The theoretical and identified damping ratios corresponding to each modal frequency are listed in Table 2, and the identified damping ratios are also close to the theoretical results. The comparison revealed excellent agreement between the modal parameter recognition method and the calculated results, confirming the effectiveness of this approach. Note that these comparisons based on the theoretical model have sufficiently verified the accuracy of the proposed identification method. Therefore, in the subsequent analysis for practical engineering applications, mode shapes and damping ratios are not further displayed and we mainly show the process of obtaining cluster centers via post-processing clustering based on the stability diagram.

### 3.3. Modal Identification Based on Numerical Modeling Results

An integrated finite element model of the wind turbine was established using Opensees, and the model excitation was white noise wave. The identification results and the calculations from Opensees were compared.

**Figure 10:**
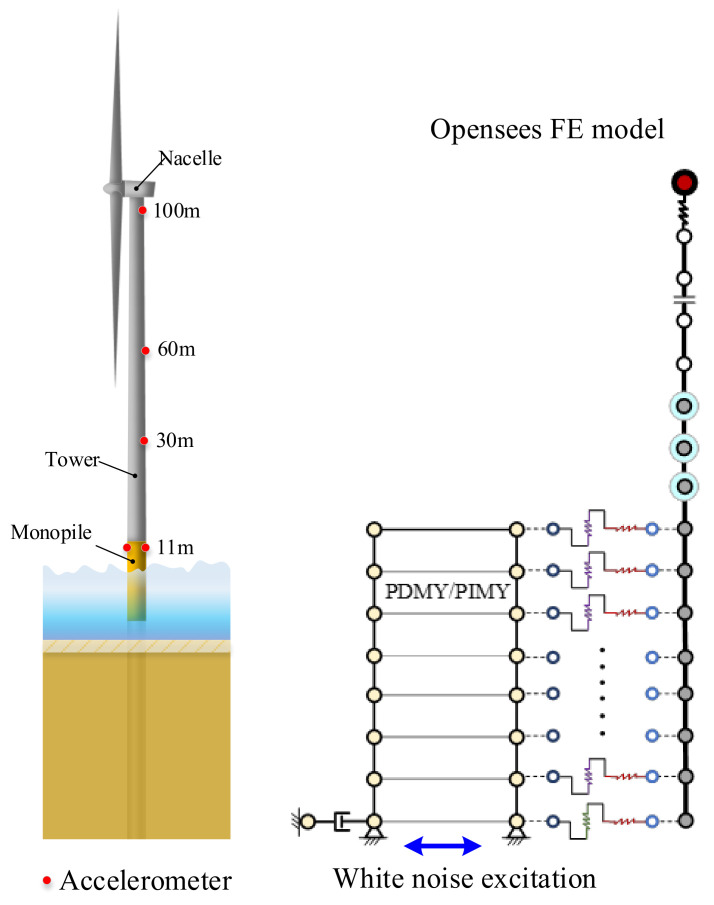
OWT monitoring setup and numerical simulation model [36,37].

**Figure 11:**
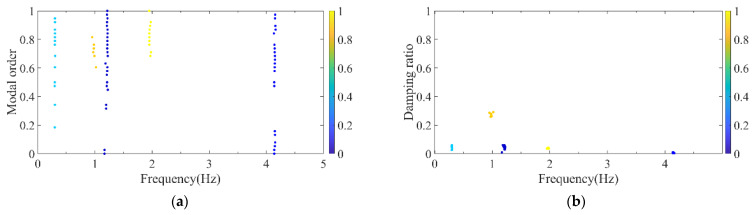
DBSCAN clustering results of OWT model. (**a**) Normalized model order and frequency; (**b**) normalized damping ratio and frequency.

**Figure 12:**
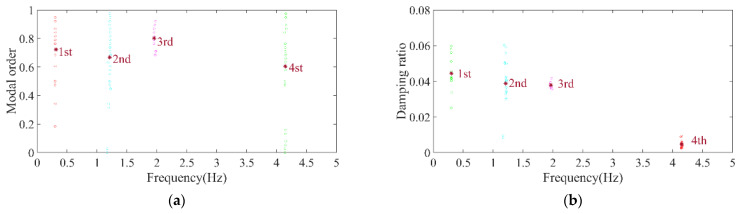
Clustering centers results of OWT model. (**a**) Normalized model order and frequency; (**b**) normalized damping ratio and frequency.

**Figure 13:**
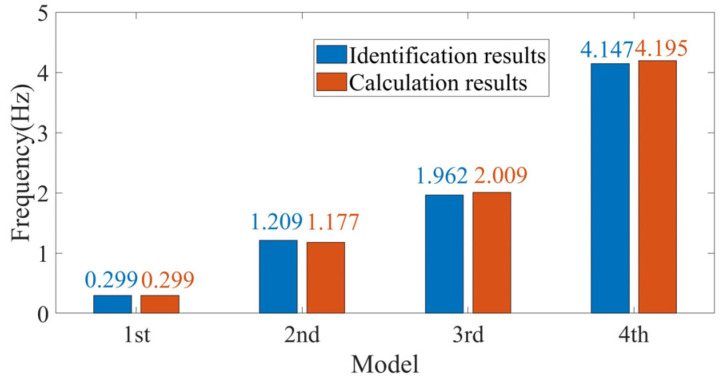
Opensees model and automatic modal identification comparation results.

The finite element model of a 3.3 MW real wind turbine was established using Opensees 2.5.0, as shown in Figure 10. This method has been previously validated in papers published by the authors’ research group [36,37]. The detailed parameters of the Opensees model are summarized in Table 3 [38]. White noise excitation was applied to the model, and acceleration data was extracted at elevations of 11 m, 30 m, 60 m, and 90 m. The modal parameters of the wind turbine were calculated using the automatic modal parameter identification method. The results of DBSCAN clustering and the K-means clustering are illustrated in Figure 11 and Figure 12. The first, second, third, and fourth modal frequencies of the wind turbine were determined to be 0.299, 1.177, 2.009, and 4.1954 Hz, respectively. A comparison with the frequencies calculated by Opensees software is presented in Figure 13, showing a difference of 0.26%, 2.71%, −2.32%, and −1.13% for the first second, third, and fourth modal frequency respectively. The mean error is 0.0318 Hz, the median error is 0.0395 Hz, and the maximum error is 0.048 Hz. This indicates the high accuracy of the modal parameter identification method.

## 4. Discussion

A structure with an open-database benchmark in Germany was adopted to identify the modal parameters using the proposed method. The frequency recognition results were compared to evaluate the performance of the proposed method. Furthermore, because of the complex operational environment of the wind turbine, which involves considerable noise, an on-site monitoring data from a 3.3 MW OWT with monopile foundation in China was employed [39]. The automatic modal parameter identification method proposed in this paper was adopted to recognize the modal parameters. Comparation between the results obtained using the proposed method and those obtained through FDD method was carried out. Additionally, the limitations of the proposed method were addressed.

### 4.1. Benchmark Dataset-Driven Comparative Analysis

**Figure 14:**
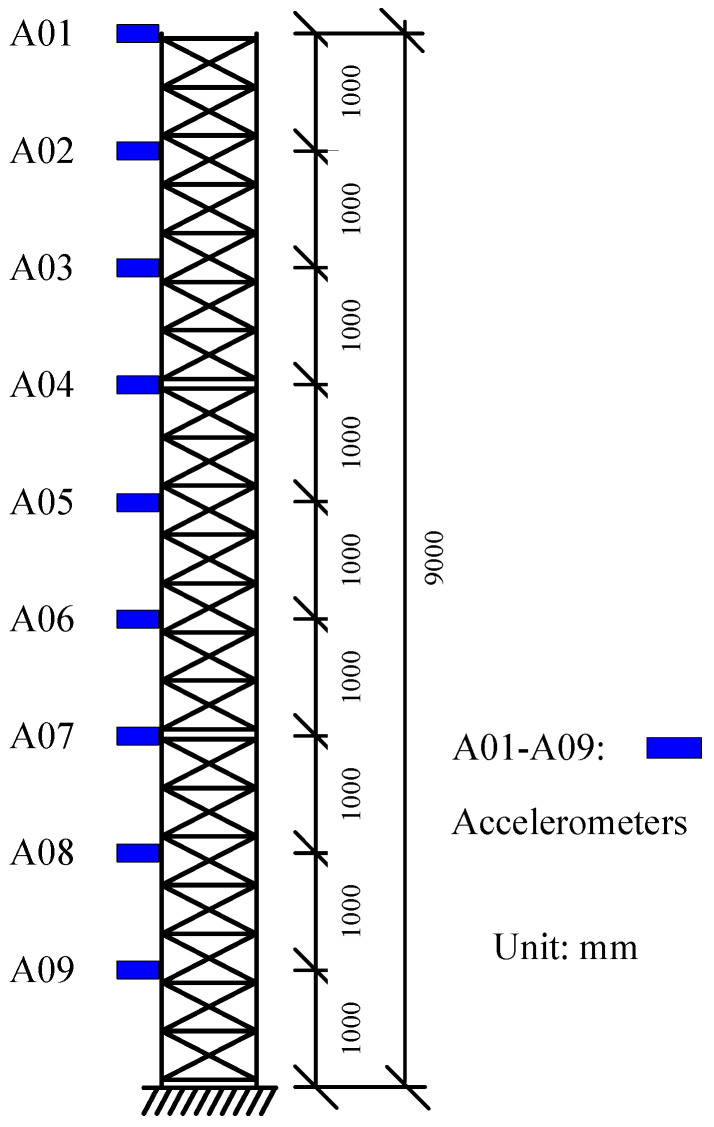
Schematic diagram of the test structure [40].

**Figure 15:**
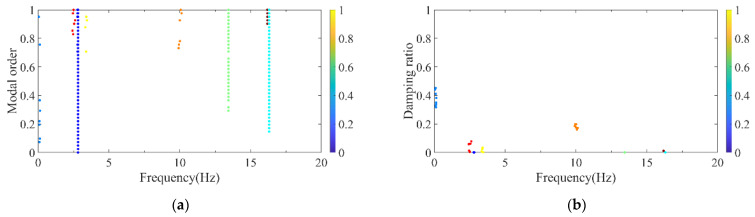
DBSCAN clustering results for the test structure. (**a**) Normalized model order and frequency; (**b**) normalized damping ratio and frequency.

**Figure 16:**
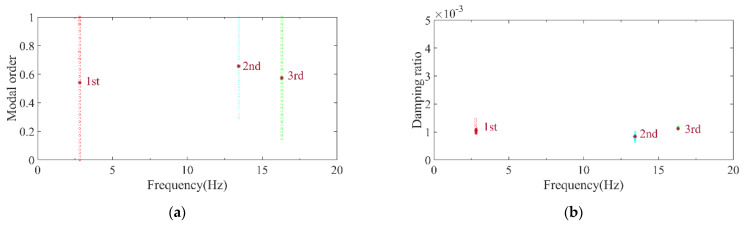
Clustering centers results for the test structure. (**a**) Normalized model order and frequency; (**b**) normalized damping ratio and frequency.

**Figure 17:**
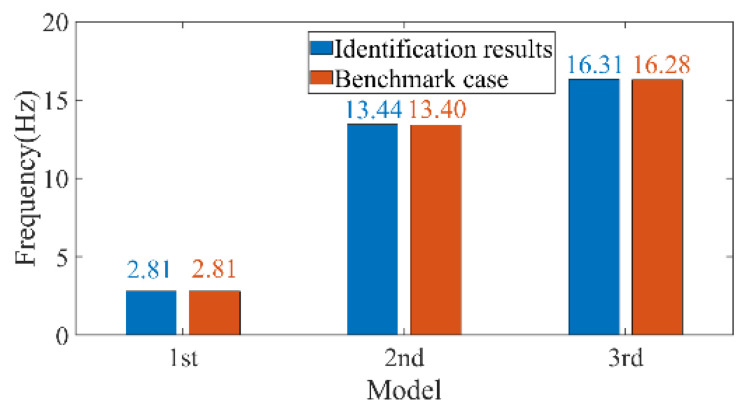
Comparation between benchmark case and automatic modal identification results.

Wernitz et al. conducted a set of experiments on structural vibration health monitoring, situated 20 km from Hanover, Germany, providing reliable benchmark data for engineers and researchers in the field [40]. The height of the test structure was 9 m and it was equipped with 18 uniaxial accelerometers, as shown in Figure 14. The relevant experimental data have been published and made publicly available on the Leibniz University website. To validate the reliability of the automatic modal parameter identification method proposed in this paper, the authors selected a segment of 10-min acceleration data in the x-direction from October 2021 among the publicly available data. The method was employed to identify modal parameters, and the results were compared with the benchmark data from [40]. Figure 15 and Figure 16 present the computed results for the first three x-direction modal frequencies, which are 2.81, 13.44, and 16.31 Hz, respectively. Figure 17 illustrates the comparison between the identified results and the benchmark case for the first three modes. Compared with the benchmark case, the frequency identified by the method proposed in this paper shows differences of 0.0%, 0.30%, and 0.18% at the first, second, and third orders respectively. The negligible discrepancies in the comparison demonstrate the reliability of the proposed method within an acceptable range.

### 4.2. Real-World OWT Implementation and FDD Method Comparison

**Figure 18:**
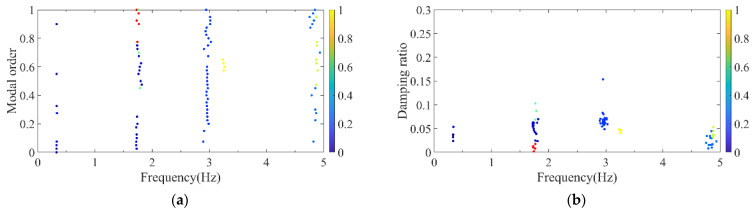
DBSCAN clustering results for monitoring data. (**a**) Normalized model order and frequency; (**b**) normalized damping ratio and frequency.

**Figure 19:**
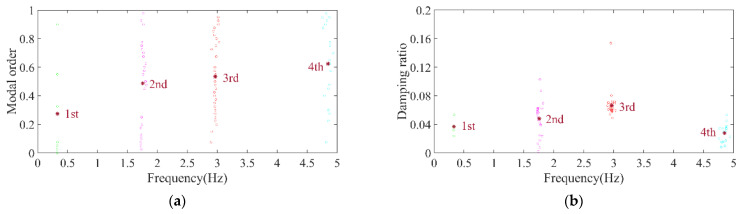
Clustering centers results for monitoring data. (**a**) Normalized model order and frequency; (**b**) normalized damping ratio and frequency.

Utilizing monitoring data from a real-word 3.3 MW OWT with monopile foundation under environmental loads, the automatic modal parameter identification method was adopted to analyze the modal parameters. Following the accelerometer arrangement scheme for the project turbine, as depicted in Figure 10, two accelerometers were placed at a height of 11 m, and one accelerometer was placed at each of the 30 m, 60 m, and 90 m elevations. The accelerometers are dual-axis accelerometers, but for the analysis in this paper, only unidirectional vibration information was utilized. The clustering results are illustrated in Figure 18 and Figure 19, with the modal frequencies being 0.329, 1.755, 2.964, and 4.847 Hz, respectively.

**Figure 20:**
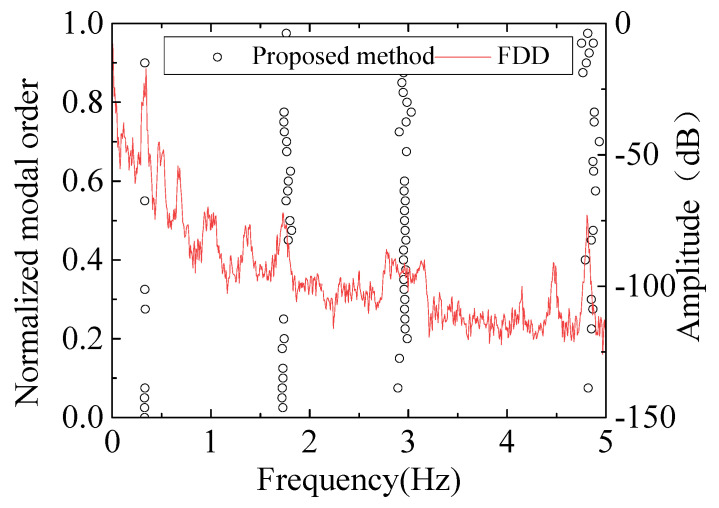
Comparation between proposed method and FDD results.

Additionally, a modal frequency comparison was made with the FDD method [41]. Initially, Fourier transform was applied to the acceleration data to extract spectral characteristics at different frequencies. Singular Value Decomposition (SVD) was then performed on the spectral matrix to identify the dominant frequency components in the frequency domain. The modal frequencies were determined by analyzing the peaks in the spectrum. The extracted principal frequencies are illustrated in Figure 20. By comparing the frequency results obtained using FDD with the final stability graph from the modal parameter automatic identification, the correctness of the latter method was further confirmed. The comparison with the FDD method is conducted to highlight the convenience, automation, and computational efficiency of the proposed approach, which focuses on robust post-processing of identified results, especially in practical engineering applications involving massive identification outputs. In contrast to the FDD method, which relies on engineers for manual visual peak identification, such a process faces notable challenges such as filtering out spurious peaks and discriminating between different modal orders in multi-peak scenarios.

**Figure 21:**
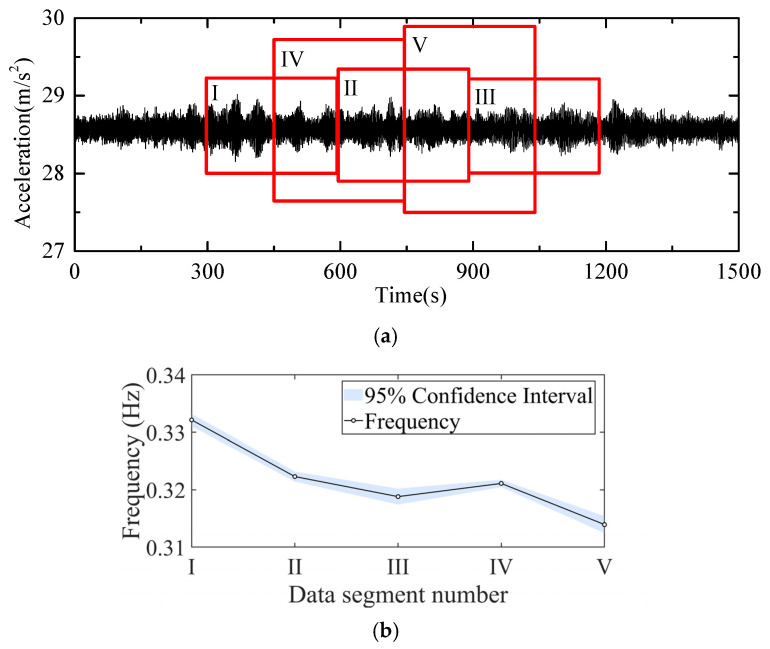
Modal frequency identification results of different data segments. (**a**) Number of segments; (**b**) 95% confidence interval.

Additionally, 20 min of acceleration monitoring data were selected, and five 5-min intervals were chosen for modal frequency calculations. Figure 21 shows segments I to V, each representing a selected 5-min dataset. The modal frequency identification results of different time periods are also shown in Figure 21. For the set obtained by first-order DBSCAN clustering, 95% confidence interval analysis was conducted. The central values of the clustering centers were identified for the first-order frequencies within each 5-min segment range from 0.3139 Hz to 0.3321 Hz, with corresponding compact 95% confidence intervals whose widths vary between 0.0013 Hz and 0.0029 Hz. This indicates that the stability analysis based on 5-min segmentation yields results of high accuracy and favorable repeatability, accompanied by an overall low degree of data dispersion. The results demonstrate the continuous stability of the outputs and the computational convenience of the method.

### 4.3. Limitations of the Proposed Methodology

The proposed methodology can effectively extract modal parameters (frequency, damping ratio and mode shape) from wind turbine vibration signals, but it still has certain limitations under actual wind turbine operating conditions, which are mainly reflected in the following aspects and will be further improved in future work.

The current methodology is mainly designed for the analysis of stable vibration signals and has limited adaptability to the time-varying characteristics of wind turbines. The operating conditions of wind turbines change continuously, including start–stop operations, variable rotational speeds, and variable loads. The wind turbine’s inherent characteristics are affected not only by its own material, shape, thickness and height, but also by overhead weight and mass eccentricity. In particular, during wind turbine operation, gyroscopic effect [42], centrifugal stiffening and centrifugal softening [43] are generated, all causing variations in the structural frequencies. The gyroscopic effect refers to the inertial property of a rotating rotor to maintain its rotational orientation, which may reduce first-order sway amplitudes. Additionally, axial force is the combination of blade gravity and centrifugal inertial force along the blade axis; it causes blade deformation and generates additional stiffness. However, existing methods are established on the assumption of time-invariant systems and cannot dynamically track real-time variations in modal parameters.

In addition, the damping of offshore wind turbines consists of multiple components, including material damping of the structure itself, structural damping arising from component connections, and hydrodynamic damping generated by the interaction between the tower, foundation and seawater. The superposition of these damping components results in an extremely complex damping mechanism, making it difficult for current modal identification methods to separate and accurately identify each damping component.

## 5. Conclusions

In this study, DBSCAN and K-means were integrated to introduce an innovative machine learning-based method for the automatic identification of modal parameters. The validity of the proposed method was then confirmed through theoretical analysis and numerical simulations. Subsequently, two monitoring scenarios were employed to calculate modal frequencies, and the results were compared with benchmark cases to evaluate the performance of the proposed method. The following conclusions were drawn.

(1) The proposed method combines the advantages of DBSCAN and K-means clustering algorithm. DBSCAN can successfully cluster noise points outside the concerned frequency range, enhancing the efficacy of the noise filter. And the number of clusters and data points within the range of interest frequencies are extracted, and any unreasonable clusters and data points are removed.
(2) The improved K-means clustering can acquire cluster centers and modal parameters automatically utilizing the number of K-means clusters from the DBSCAN clustering process. It optimizes the traditional clustering algorithm by eliminating the need to specify the number of clusters.
(3) The verification and comparative case study outcomes demonstrate that the proposed method possesses the accuracy and stability required for automated modal parameter identification, making it applicable to the modal characterization of OWTs. The implementation of this approach will contribute to ensuring the secure operation and maintenance of OWTs.

Despite the effectiveness and automation of the proposed method in modal identification post-processing, several limitations remain and can be further improved in future work. First, the current clustering parameters are determined based on empirical analysis and data distribution; therefore, adaptive parameter optimization will be explored to enhance the generalization performance across different structures and measurement scenarios. In addition, the entire post-processing workflow will be extended to enable automatic figure generation and standardized report output, further improving its engineering practicability. Furthermore, advanced artificial intelligence techniques [44] will be incorporated to achieve adaptive parameter optimization and intelligent fault identification, thereby enhancing the robustness, generalization, and intelligence of modal identification under complex operational conditions.

## Figures and Tables

**Table 1:** Achievements and remaining gaps for current methods.

Combined Methods	Key Achievements	Critical Limitations
SSI and Hierarchical Clustering [27]	Hierarchical clustering can automatically cluster stable poles from SSI without presetting cluster number in advance and effectively eliminates false modes.	It has high computational cost and its clustering result is easily affected by distance threshold selection.
SSI and K-means [19,28]	K-means clustering effectively groups stable poles and eliminates false modes in SSI results.	It requires subjective cluster number selection, sensitivity to outliers, and poor performance for closely spaced modes.
SSI and Spectral Clustering [29]	Spectral clustering achieves global optimal classification of stable poles in SSI and effectively distinguishes true and false modes even for closely spaced modes.	It suffers from high computational complexity and relies on manual selection of similarity measurement parameters
SSI and Fuzzy Clustering [20,30]	Fuzzy clustering realizes soft division of stable poles and improves the adaptability to discrete modal data in SSI.	It relies on subjective selection of membership parameters and has high computational complexity.
SSI + DBSCAN [18,21,22,23,24]	DBSCAN can interpret the stabilization diagram from SSI using user-defined parameters.	It cannot automatically identify the cluster centers to extract modal parameter information.

**Table 2:** The theoretical and identified damping ratios.

Order	Theoretical Value/%	Identified Result/%	Error/%
1	3.18	2.78	12.73
2	4.64	4.21	9.33
3	6.92	7.21	−4.13

**Table 3:** Numerical model parameters [38].

Model Component	Settings	Values	Modeling Techniques
RNA	Rotor diameter (m)	140.5	Lumped mass
	Mass (t)	218.28	
Tower	Hub height above MSL (m)	92	Displacement-based beam-column
	Tower height (m)	78	
	Tower diameter (m)	3.3–5.5	
	Tower wall thickness (m)	0.014–0.038	
	Elasticity modulus (kPa)	2.10 × 10^8^	
	Mass density (kg/m3)	8.50 × 10^3^	
Pile	Monopile length (m)	70	Displacement-based beam-column
	Length above MSL (m)	12	
	Water depth (m)	3	
	Embedded length (m)	48	
	Monopile diameter (m)	5.5	
	Monopile wall thickness (m)	0.050	
Soil	Scour depth(m)	6	
	Pile–soil interaction		Nonlinear hysteretic p-y model for horizontal interaction, and TzSimple1 and QzSimple1 for vertical interaction
Damping	Damping ratio η	0.02	Rayleigh damping
Boundary condition	Lysmer–Kuhlemeyer viscous boundary	-	Dashpots

## Data Availability

Dataset available on request from the authors.

## Abbreviations

The following abbreviations are used in this manuscript:

OWTs	offshore wind turbines
SSI-data	data-driven stochastic subspace identification
DBSCAN	Density-Based Spatial Clustering of Applications with Noise
SSI	stochastic subspace identification
FDD	Frequency Domain Decomposition
MVC	modal validation criteria
Fre	frequency difference
Dam	damping ratio difference
MAC	mode assurance criterion
MPC	modal phase coherence
MPD	mean phase difference
SVD	Singular Value Decomposition
